# Global actions of nicotine on the striatal microcircuit

**DOI:** 10.3389/fnsys.2013.00078

**Published:** 2013-11-06

**Authors:** Víctor Plata, Mariana Duhne, Jesús Pérez-Ortega, Ricardo Hernández-Martinez, Pavel Rueda-Orozco, Elvira Galarraga, René Drucker-Colín, José Bargas

**Affiliations:** División de Neurociencias, Instituto de Fisiología Celular, Universidad Nacional Autónoma de MéxicoMexico City, Mexico

**Keywords:** striatal microcircuit, nicotine, nicotinic receptors, GABAergic interneurons

## Abstract

The question to solve in the present work is: what is the predominant action induced by the activation of cholinergic-nicotinic receptors (nAChrs) in the striatal network given that nAChrs are expressed by several elements of the circuit: cortical terminals, dopamine terminals, and various striatal GABAergic interneurons. To answer this question some type of multicellular recording has to be used without losing single cell resolution. Here, we used calcium imaging and nicotine. It is known that in the presence of low micromolar N-Methyl-D-aspartate (NMDA), the striatal microcircuit exhibits neuronal activity consisting in the spontaneous synchronization of different neuron pools that interchange their activity following determined sequences. The striatal circuit also exhibits profuse spontaneous activity in pathological states (without NMDA) such as dopamine depletion. However, in this case, most pathological activity is mostly generated by the same neuron pool. Here, we show that both types of activity are inhibited during the application of nicotine. Nicotine actions were blocked by mecamylamine, a non-specific antagonist of nAChrs. Interestingly, inhibitory actions of nicotine were also blocked by the GABA_A_-receptor antagonist bicuculline, in which case, the actions of nicotine on the circuit became excitatory and facilitated neuronal synchronization. We conclude that the predominant action of nicotine in the striatal microcircuit is indirect, via the activation of networks of inhibitory interneurons. This action inhibits striatal pathological activity in early Parkinsonian animals almost as potently as L-DOPA.

## Introduction

The striatal microcircuit is composed of projection neurons and different classes of interneurons (e.g., Kawaguchi, 1993; Tepper and Bolam, 2004; Tepper et al., 2010). This circuit receives inputs from the cortex, the substantia nigra compacta (SNc) and the thalamus, among others, being a main entrance to the basal ganglia, a system of nuclei which encodes movement, associative learning, and procedural memory (Cools, 1980; DeLong, 1981; Aosaki et al., 1994; Kreitzer and Malenka, 2008; Do et al., 2012).

Although striatal projection neurons (SPNs) have low rates of basal firing in control conditions (e.g., Mink, 2003), uncorrelated excitatory drives such as N-Methyl-D-aspartate (2–5 μM NMDA) induces correlated neuronal activity in the control striatal microcircuit *in vitro* (Carrillo-Reid et al., 2008); similar to that produced during movement *in vivo* (Vautrelle et al., 2009). This activity consists in moments of recurrent and spontaneous synchronization in the firing of different neuron pools. This synchronous activity is alternated among the different neuron pools, generating the appearance of determined sequences, some of them being reverberant sequences or cycles (Carrillo-Reid et al., 2008; 2009a). These dynamics have been shown to be modulated by transmitters acting through G-protein coupled receptors and signaling pathways such as those activated by dopamine (DA) and acetyl-choline (ACh) (2009a; Carrillo-Reid et al., 2011).

On the other hand, when deprived of DA supply, as in animal models of Parkinson's disease (PD), the striatal circuitry also generates a profuse spontaneous and synchronized activity without the addition of NMDA or any other excitatory drive. However, this pathological activity induced by DA-depletion differs from that found in control tissue: it is characterized by the loss of sequential activity and alternating dynamics. Almost all activity becomes generated by the same neuron pool with recurrent synchronization, resembling the repetitive oscillations found in Parkinsonian subjects (Jáidar et al., 2010). Here, we show that both control (with NMDA) and Parkinsonian activities are globally suppressed by nicotine administration.

The ACh present in the striatal microcircuit is released by local cholinergic interneurons and is the highest in any brain region together with the levels of choline acetyl-transferase, and choline-esterase (Mesulam et al., 1992; Contant et al., 1996; Goldberg et al., 2012). Cholinergic interneurons are autonomous pacemakers and ACh release is continuous and dynamic, thus, producing a varying tonic level of ACh in the whole striatum according to demand (Bennett and Wilson, 1999; Goldberg and Wilson, 2005).

The majority of the neurons (>90%) in the striatal circuit are SPNs which respond to ACh via muscarinic G-protein coupled receptors (Galarraga et al., 1999; Alcantara et al., 2001; Yan et al., 2001; Zhang et al., 2002). Known actions of these muscarinic receptors are facilitatory due in part to suppression of K^+^-outward currents, directly or indirectly (Howe and Surmeier, 1995; Gabel and Nisenbaum, 1999; Galarraga et al., 1999; Lin et al., 2004; Olson et al., 2005; Pérez-Burgos et al., 2008; Pérez-Rosello et al., 2005; Shen et al., 2005).

However, much less is known about the nicotinic receptors present in this circuit (Goldberg et al., 2012). It is known that nAChRs are present in striatal dopaminergic terminals and promote DA release (e.g., Wonnacott et al., 2000; Grady et al., 2007; Keath et al., 2007; Livingstone and Wonnacott, 2009; Xiao et al., 2009; Cachope et al., 2012; Threlfell et al., 2012). It is also known that they are present in the terminals of cortical afferents and promote glutamate release (e.g., Marchi et al., 2002; Zhang and Warren, 2002; Campos et al., 2010). Finally, they are present in striatal GABAergic interneurons promoting GABA release that inhibits projection neurons (Koós and Tepper, 2002; Wilson, 2004; Kreitzer and Malenka, 2008; Livingstone and Wonnacott, 2009; Xiao et al., 2009; English et al., 2011; Ibáñez-Sandoval et al., 2011; Luo et al., 2013). Each of these actions has been studied directly and separately in cell-focused studies. However, it is not known which of them predominate in the microcircuit as a whole during nAChrs agonist administration.

Note that, if actions on glutamate afferents were predominant, we should see an enhancement of activity similar to that produced by NMDA alone, and a summation of effects would be evident. On the other hand, if the release of DA were the main action, mixed effects would appear: some interneurons are activated by DA, although their GABA release may be inhibited, being hard to foretell what is the net result (Bracci et al., 2002; Centonze et al., 2003). Activation of interneurons may enhance inhibition due to cholinergic activation. But at the network level, SPNs may be inhibited or excited by DA (e.g., Kiyatkin and Rebec, 1996), and both classes of DA-receptors, D_1_ and D_2_, increase synchronous firing (Carrillo-Reid et al., 2011). In addition, in the DA-depleted circuit, activity increases pathologically (Jáidar et al., 2010) while collateral inhibition is decreased (Taverna et al., 2008). In summary, a host of mixed and parallel actions makes hard to foretell what would be the global action of nicotinic receptors activation on the striatal microcircuit.

The importance of answering this question resides in the suspected neuro-protective action of nicotine in the prevention and development of PD (e.g., Costa et al., 2001; Quik et al., 2007; Kawamata et al., 2012). Although this postulate is still controversial (García-Montes et al., 2012), the hypothesis has its origin in epidemiological studies that claim less incidence of PD in smokers (Gorell et al., 1999; Herman et al., 2001; Driver et al., 2009), and in clinical studies that claim improvements of motor and cognitive symptoms in PD patients subjected to nicotine analogs (Fagerström et al., 1994; Villafane et al., 2007). From these arguments comes the importance of knowing with certainty what would be the end result of administering a tonic level of a nAChrs agonist in the striatal circuit, which in the present case is seen as a neuronal population of diverse elements capable to generate assembly dynamics (Carrillo-Reid et al., 2008) and involved in the generation of PD signs and symptoms.

## Materials and methods

### Slice preparation

Corticostriatal slices (300 μm) were obtained from PD20-40 male mice as previously described (Vergara et al., 2003). Animal experimentation followed the National Institutes of Health Guide for Care and Use of Laboratory Animals and the National University of Mexico guidelines. Slices were obtained with ice-cold saline (4°C) containing in mM: 123 NaCl, 3.5 KCl, 1 MgCl_2_, 1.5 CaCl_2_, 26 NaHCO_3_, and 11 glucose (saturated with 95% O_2_ and 5% CO_2_). Slices remained in saline at room temperature (21–25°C) for at least 1 h before the experiments.

### Optical recordings of neuronal populations with single cell resolution

Slices were incubated in the dark with 10 μ M of the calcium indicator fluo-8 AM for about 20 min (Tef Labs, Austin, TX) in saline containing 0.1% dimethylsulphoxide (DMSO), equilibrated with 95% O_2_ and 5% CO_2_. We used an upright microscope equipped with a 10X, 0.95 NA water-immersion objective (E600FN Eclipse, Nikon, Melville, NY). To observe the changes in fluorescence we delivered pulses at 488 nm (50–100 ms exposure) to the preparation with a Lambda LS illuminator (Sutter instruments, Novato CA) connected to the microscope via fiber optics. The image field was 800 × 600 μm in size. Short movies (~180 s = epoch) were acquired at time intervals of 5–20 min during ≥60 min with a cooled digital camera (SenSys 1401E, Roper Scientific, Tucson, AZ) at 100–250 ms/frame.

Neurons active during the experiment (30 to 300 depending on number of epochs and age of animals; Carrillo-Reid et al., 2008) were identified due to their spontaneous calcium transients. They were recorded in control saline or during the application of NMDA and/or nicotine with or without ionotropic channel blockers such as: 6-cyano-2, 3-dihydroxy-7-nitro-quinoxaline disodium salt (10 μM CNQX), D-(-)-2-amino-5-phosphonovaleric acid (50 μM APV), bicuculline (10 μM) or gabazine (10 μM) (Sigma-Aldrich-RBI, St. Louis, MO). Stock solutions were prepared before experiments and added to the recording chamber in the final concentration indicated.

### Image processing

Neurons active in the field of view were selected automatically by a custom made program written in the LabView™ programming environment. The program processes the image sequence obtaining the fluorescence signals originated from action potentials discharge (Carrillo-Reid et al., 2008; 2009a). Briefly, a two dimensional coordinate was assigned for each cell. Each neuron was numbered and its precise location in the field of view was known. Calcium transients represent changes in fluorescence: (F_i_-F_o_)/F_o_, where F_i_ denotes the fluorescence intensity at any frame and F_o_ denotes the basal fluorescence of each neuron. As it has been reported (Carrillo-Reid et al., 2008, 2009b; Jáidar et al., 2010) the first time derivative of the calcium transient reflects the time of electrical discharge of striatal neurons (>2.5 times the standard deviation of the noise value), in this way, the electrical activity of each neuron in the field of view could be followed along the experiment.

We constructed binary matrixes with the activity of dozens of neurons recorded simultaneously (raster plots). In each matrix, each row denotes an active neuron (numbered), while each column represents a time frame when an image was taken. Time axis represents the total number of frames making each movie converted into a minutes scale. For analysis, we considered calcium transients elicited by neurons only. Signals from neurons are much faster than signals from glial cells (Ikegaya et al., 2004; Sasaki et al., 2007; Carrillo-Reid et al., 2008). To visualize electrical activity from several active neurons, the binary matrix was plotted as a raster plot where neuronal firing is represented by dots. The activity histogram, shown below the raster plot, illustrates population activity of all neurons recorded during an experiment; it is obtained by the addition of all active neurons in each frame. Each spontaneous peak of synchronous activity denotes a pool of several neurons firing together (or having closely correlated activity). Note that each peak of synchronization is a neuronal column vector. Different colors denote that neuronal vectors are composed of different neuron pools.

### Statistical methods

Statistically significant peaks of co-active neurons were vectorized identified and counted (Carrillo-Reid et al., 2008), therefore, the level of correlated firing in the network can be quantitatively assessed and statistically compared. To assess the probability that a given peak of synchronization had appeared by chance, the points of the same matrix (raster plot) were used for MonteCarlo simulations with 10,000 replications. Thus, a level of significance is marked (dashed line) for all activity histograms. All the peaks of synchronization denoted by colors surpassed the significance level (*P* < 0.05). In control conditions without NMDA there are no significant peaks of synchronization. However, they appear in control tissue after adding NMDA. In addition they appear without NMDA when the tissue is depleted of dopamine (DA-depleted). If a treatment suppress NMDA-induced activity or DA-depletion induced activity, significant peaks of synchronization may go away in the activity histogram and the matrix only contains black dots denoting a decrease of network activity.

The sequence of peaks of synchronization in the activity histogram denotes the activity of the microcircuit along time, that is, a sequence of neuron pools synchronizing their firing and alternating their activity with other neuron pools. This type of circuit behavior has been called assembly dynamics (e.g., Carrillo-Reid et al., 2008). To know whether synchronization increased or decreased after a given treatment, the number of neuronal vectors was counted in each of several image sequences (epochs) at different times in the same experiment. Mean of averages from different slices were lumped together and a free-distribution statistic was employed (Mann-Whitney's U) for comparison. A Wilcoxon *T*-test was used to compare the same slice under different conditions. Note that each 3 min epoch commonly has dozens of individual cells. For comparison of epochs from different slices (Figure 3) the Kruskal-Wallis one way analysis of variance was used with *post hoc* Dunn tests.

These peaks of synchronization denote neuronal vectors that represent microcircuit activity in a multidimensional space, where the number of dimensions is given by the total number of active cells in each vector. Vectorization of network activity along the experiment allows the searching of recurrent patterns of activity, i.e., vectors being active repeatedly or different vectors alternating their activity (Schreiber et al., 2003; Ikegaya et al., 2004; Brown and Williams, 2005; Carrillo-Reid et al., 2008). The set of all these vectors connected by arrows represent the transitions between network states. To know whether the same vectors are active several times, similarity maps were constructed and all possible vector pairs were compared along time. The similarity index between any pair of vectors is defined by their normalized scalar product (Sasaki et al., 2007; Carrillo-Reid et al., 2008, 2009b): the cosine of the angle between the compared vectors. High similarity between vectors means that the activity of almost the same cells (same neuron pool) generated them at different times (Schreiber et al., 2003; Carrillo-Reid et al., 2008; 2009a; Jáidar et al., 2010). Different vectors (different neuron pools) are denoted by different colors in raster plots, activity histograms and locally linear embedding (LLE) graphs.

The method used to detect the dynamics of network states from multidimensional vectors has been published (Carrillo-Reid et al., 2008, 2009b; Jáidar et al., 2010). Briefly, dimensionality of population vectors representing network states was reduced by LLE, a dimensionality reduction technique that preserves the structure of non-linear multidimensional data (Roweis and Saul, 2000; Brown and Williams, 2005; Carrillo-Reid et al., 2008). Vectors are then projected into a two dimensional space. As a result, it is possible to visualize clusters of data points representing the recurrence of similar vectors with similar pools of neurons (network states) alternating their activity. Their sequences of activation may follow cycles or reverberation denoted by arrows (Schreiber et al., 2003; Sasaki et al., 2007; Carrillo-Reid et al., 2008, 2009b; Jáidar et al., 2010). To choose the optimal number of network states we used hard and fuzzy clustering algorithms and the Dunn's index as a validity function (Bezdek and Pal, 1998; Sasaki et al., 2007; Carrillo-Reid et al., 2008, 2009b).

Global neuronal activity over a given time was also represented by cumulative distributions of all cell activity in a given epoch. The rates of accumulation were approximated with *ad hoc* linear regressions. Their average slopes ± their estimation errors were compared for significant differences with non-paired Student's *t* tests, experiment by experiment. Average significance is reported. In addition, for sample comparisons of these parameters we used Wilcoxon's T statistic for paired samples and Mann-Whitney U statistic for non-paired samples (Plata et al., 2013).

### The 6-OHDA hemi-parkinsonian model

Hemiparkinsonian animals, rats or BAC-mice, were obtained as previously reported (Jáidar et al., 2010). Briefly, animals were anesthetized with ketamine (85 mg/kg, i.p.) plus xylazine (15 mg/kg, i.p.) while immobilized on a stereotactic frame. Each animal received a unilateral injection of 6-OHDA (8 μg in 0.2 μl with 0.2 mg/ml of ascorbic acid) 0.1 μl/1 min in the substantia nigra pars compacta (SNc coordinates: 3.8 mm caudal, 1.8 mm lateral to bregma, and 7.1 mm ventral to the skull surface in rats and 2.6 mm caudal, 0.7 mm lateral to bregma and 4.5 mm ventral to the skull surface in post natal day 21 mice).

As described before (López-Huerta et al., 2012), the degree of DA deprivation was tested provoking turning behavior 8 days after the surgery using automated rotometers and amphetamine (4 mg/kg, i.p.). Because these protocol has been extensively reported in several occasions (6-OHDA model; e.g., López-Huerta et al., 2012), here will not be reported in the Results, but only as a method to obtain hemiparkinsonian animals. Left and right full body turns were recorded for 90 min by a home-made computerized monitor. Animals showing >500 turns ipsilateral to the injected side were considered for further experiments.

## Results

### Cell assembly dynamics in the striatal microcircuit

**Figure 1:**
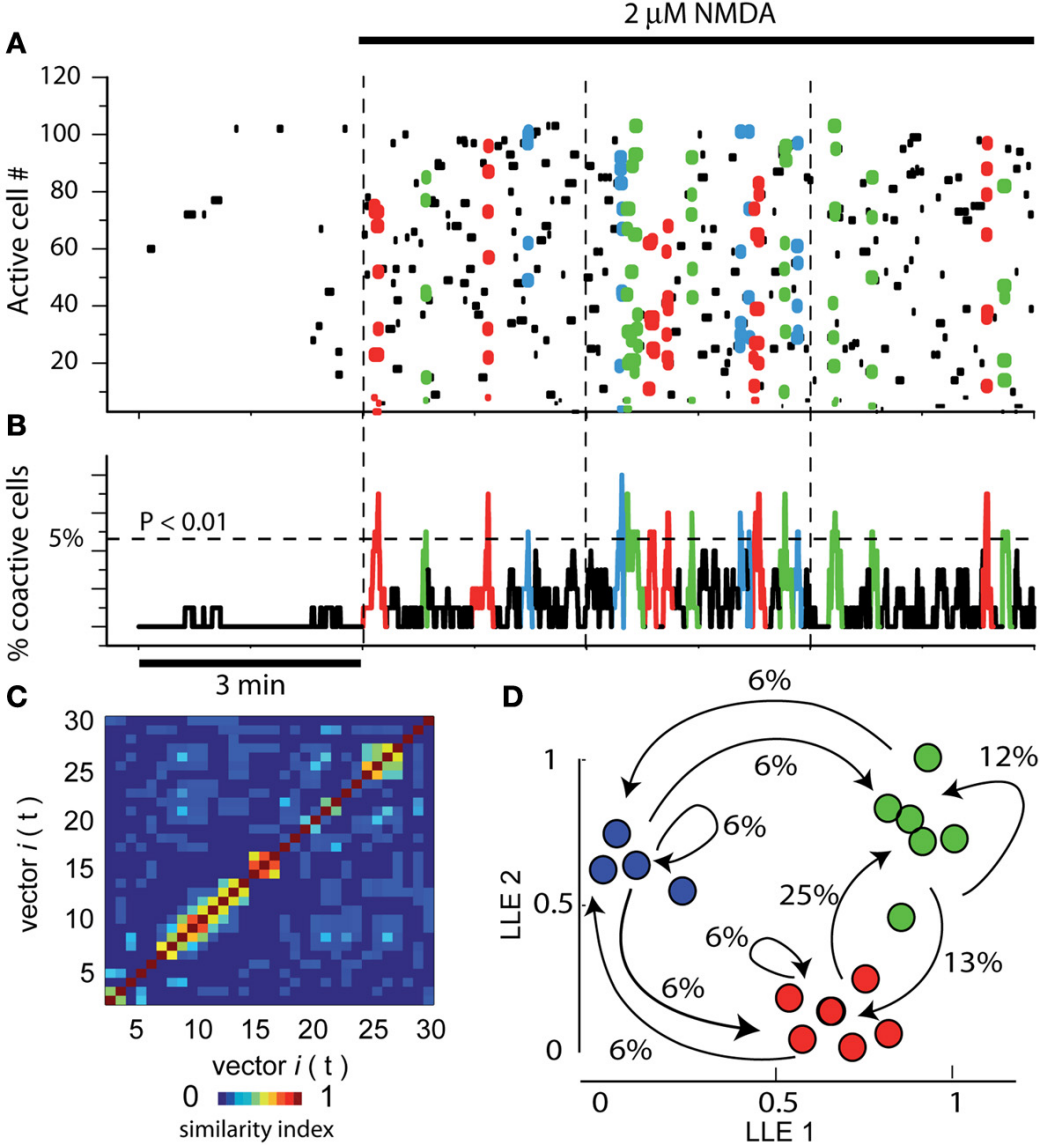
**Assembly dynamics in the control striatal circuitry after NMDA. (A)** Raster plot showing the simultaneous activity of >100 neurons in a striatal slice using calcium-imaging. Each row in the matrix represents the activity of a single neuron across the series of images (columns). Left epoch shows 3 min of activity in control striatal tissue without NMDA. Note scarce activity and the absence of peaks of synchronization. In the next three epochs, separated by dashed vertical lines, 9 min of activity are shown after adding 2 μM NMDA into the bath saline (denoted by horizontal black line on top). Note that many more dots, some of them colored, populate the matrix. Colored dots denote the synchronized activity of several neurons in a given column or neighboring columns (a neuron pool is then represented as a column vector). Note that different neuron pools produce the circuit activity along time. **(B)** Activity histogram at bottom is the summed neuronal activity (multicellular activity) from the raster plot above column by column (frame by frame in a given movie). The dashed line shows the level of significance for the spontaneous peaks of synchronization denoted by colors (obtained by Monte Carlo simulations; statistically significant neuronal vectors; *n* = 6 slices). **(C)** A similarity index matrix compares each vector with all others along time: a patchy appearance shows that similar vectors were in charge of activity. **(D)** Dimensionality reduction using locally linear embedding (LLE) shows neuronal vectors projected in a two dimensional space with no units. Similar vectors grouped together (denoted by different colors) give raise to network states. The transitions among network states are denoted by arrows. Percentages give the probability to leave a given state. Colored dots and arrows represent the sequential activity of the circuit, that is, pools of neurons synchronized their firing and pass their activity from one pool of neurons to the other: cell assembly dynamics (Carrillo-Reid et al., 2008). Note reverberant trajectories in the sequence. Here and in the next figures, epochs (times of continuous image series) are separated by vertical dashed lines.

Figure 1A illustrates a raster plot or matrix showing the activity of >100 neurons in a field of view within a striatal slice in control conditions. Each row in the plot represents the electrical activity of one neuron across a series of images (columns) recorded by means of calcium-imaging using fluo-8 (see Materials and Methods). Leftmost frame shows 3 min of activity (an epoch) in control striatal tissue without NMDA: note the scarcity of spontaneous activity and the absence of significant peaks of synchronization. In the next three frames (3 epochs 3 min each) it is shown an increase in activity involving dozens of striatal individual neurons (single cell resolution) after adding 2 μM NMDA into the bath saline. All three epochs display the NMDA-induced activity. Colored dots denote neurons firing together and belonging to a pool of neurons. Different colors denote different neuron pools. The activity of these neurons is vectorized (column vectors). Note that neuronal vectors alternate their activity along time, that is, the network transits from one set of neurons to the other as indicated by different colors.

Activity histogram at bottom represents (Figure 1B) the summed neuronal activity of the matrix above, column by column, over time. Therefore, it represents a multicellular or population recording but with the possibility of locate and count each of the cells composing the peaks of synchronization (Carrillo-Reid et al., 2008; 2009a). The dashed horizontal line shows the level of statistical significance for the peaks of synchronization (obtained by Monte Carlo simulations, see above). Note that several peaks of neuronal synchronization, denoted by colors, cross this level, indicating that sets neurons synchronize their firing significantly and spontaneously and then alternate their activity with other synchronous neuron pools. Similarity index (Figure 1C) compares each neural vector with all others over time: a patchy appearance shows that similar and several vectors were in charge of activity through the time. Dimensionality reduction of neuronal vectors using LLE shows the same vectors projected into a two dimensional space with no units (Figure 1D; Carrillo Reid et al., 2009a). Similar vectors group together (denoted by different colors) indicating various (3) network states in the circuit. Transitions between network states follow determined sequences or trajectories (arrows). Percentages give the probability of leaving a given network state. In this way, we can say that the group of neurons in the field of view shows cell assembly dynamics (Carrillo-Reid et al., 2008; 2009a). Note that in the control without NMDA (leftmost epoch in Figure 1A) there is no assembly dynamics. In the next three Figures, control activity indicates activity in the presence of 2 μM NMDA, that is, cell assembly dynamics or circuit activity. It is in this activity where we tested the global action of nicotine in the striatal circuitry.

### Global action of nicotine in the activity of the striatal microcircuit is inhibitory

**Figure 2:**
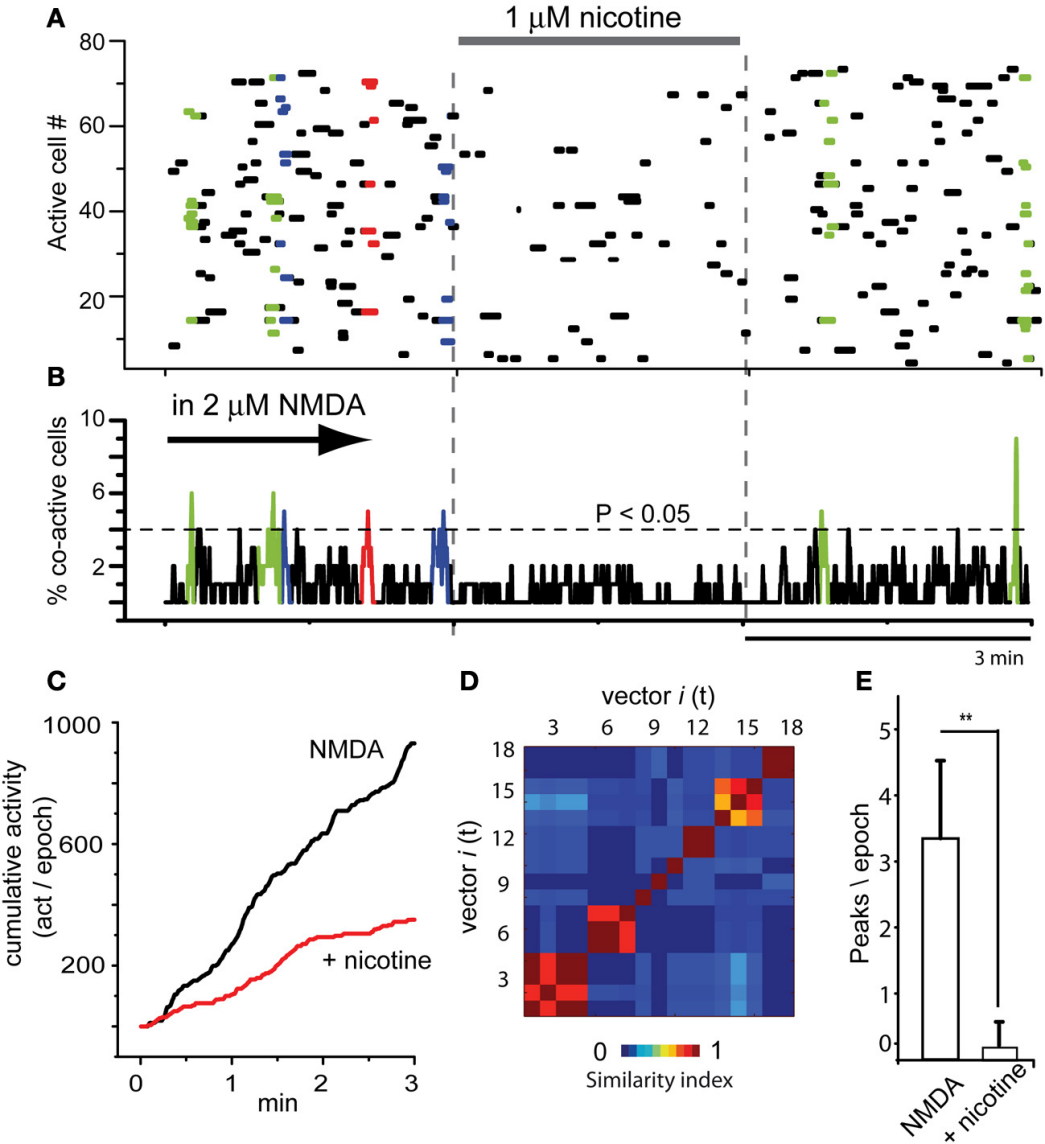
**Global actions of nicotine in the striatal circuit are inhibitory. (A)** Raster plot illustrating three brief epochs (3 min each) taken from larger movies (as in Figure 1A). Left epoch: the simultaneous activity of 80 neurons in the continuous presence of NMDA (2 μM; denoted by an arrow, cf., Figure 1). The arrow denotes that NMDA is maintained all the time during this experiment. Colored dots show neuronal vectors indicating spontaneous synchronous activity of neuron pools. Middle epoch: nicotine (1 μM) is added to the bath saline in the presence of NMDA. Note that activity is decreased and the absence of colored dots indicates that spontaneous events of synchronization are now absent. Right epoch: a partial nicotine washing off, two significant peaks of synchronization appear indicating that nicotine actions were reversible. **(B)** Activity histogram displaying multicellular activity. Significant peaks of synchronization (colored) appear during NMDA but not after addition of nicotine even if NMDA is present. **(C)** Cumulative activity taken from histogram in **(B)** (addition of all bars in the histogram over time in a given epoch) shows that rate of accumulated activity was significantly higher in the absence of nicotine. Nicotine lowered the rate of cumulative activity significantly in all slices tested (*n* = 6 slices; *n* = 3 epochs per slice were taken). **(D)** Matrix of vectors similarity along time, without nicotine. **(E)** Histogram compares the appearance of peaks of synchronization per 3 min epoch in NMDA and in NMDA plus nicotine: it is clear that nicotine significantly abolished NMDA-induced synchronous activity. ^**^P < 0.025.

Raster plot in Figure 2A illustrates three epochs (3 min each separated by dashed vertical lines): the left epoch shows an activity similar to that described in Figure 1A in the continuous presence of 2 μM NMDA (arrow; NMDA was present all the time during this experiment): this activity reveals significant peaks of synchronization in the activity histogram below, the manifestation of assembly dynamics (Figure 2B colored). In NMDA presence, addition of 1 μM nicotine to the bath saline (middle epoch) drastically reduced the level of neuronal activity (horizontal bar indicates time of nicotine exposure). Nicotine reduced most circuit activity: less dots, absence of significant peaks synchronization. Note that the actions of nicotine were reversible upon washing (right epoch). Histogram of multicellular activity (Figure 2B; note significance level indicated by a dashed horizontal line) shows spontaneous and significant peaks of synchronization only in the presence of NMDA alone. Addition of nicotine abolished the peaks of synchronized activity even in the presence of NMDA (Figure 2B). Nonetheless, after nicotine was washed off, the peaks of synchronization begin to return (right epoch). By summating all activity from histogram in Figure 2B over time (all bars in the histogram for each epoch), we obtained a graph of cumulative cell activity (Figure 2C). It shows that total circuit activity in the presence of NMDA is much higher (*ad hoc* fitting of straight lines where slopes become the rate of accumulation along time ± estimation error). An average of a sample of experiments yields 479 ± 2 (act/min), which decays to 190 ± 1.5 (act/min) during nicotine (*n* = 6 slices; *P* < 0.005). Figure 2D shows the similarity matrix when nicotine is not present reassuring that assembly dynamics was present during NMDA. In addition, we counted the number of significant peaks of synchronization per epoch in the presence of NMDA and during nicotine in the continuous presence of NMDA (Figure 2E): an average of 3.3 ± 1.2 peaks/epoch in the control (NMDA) vs. 0.16 ± 0.4 peaks/epoch in the presence of nicotine (*n* = 6 slices; *n* = 3 epochs per slice; ^**^P < 0.025).

**Figure 3:**
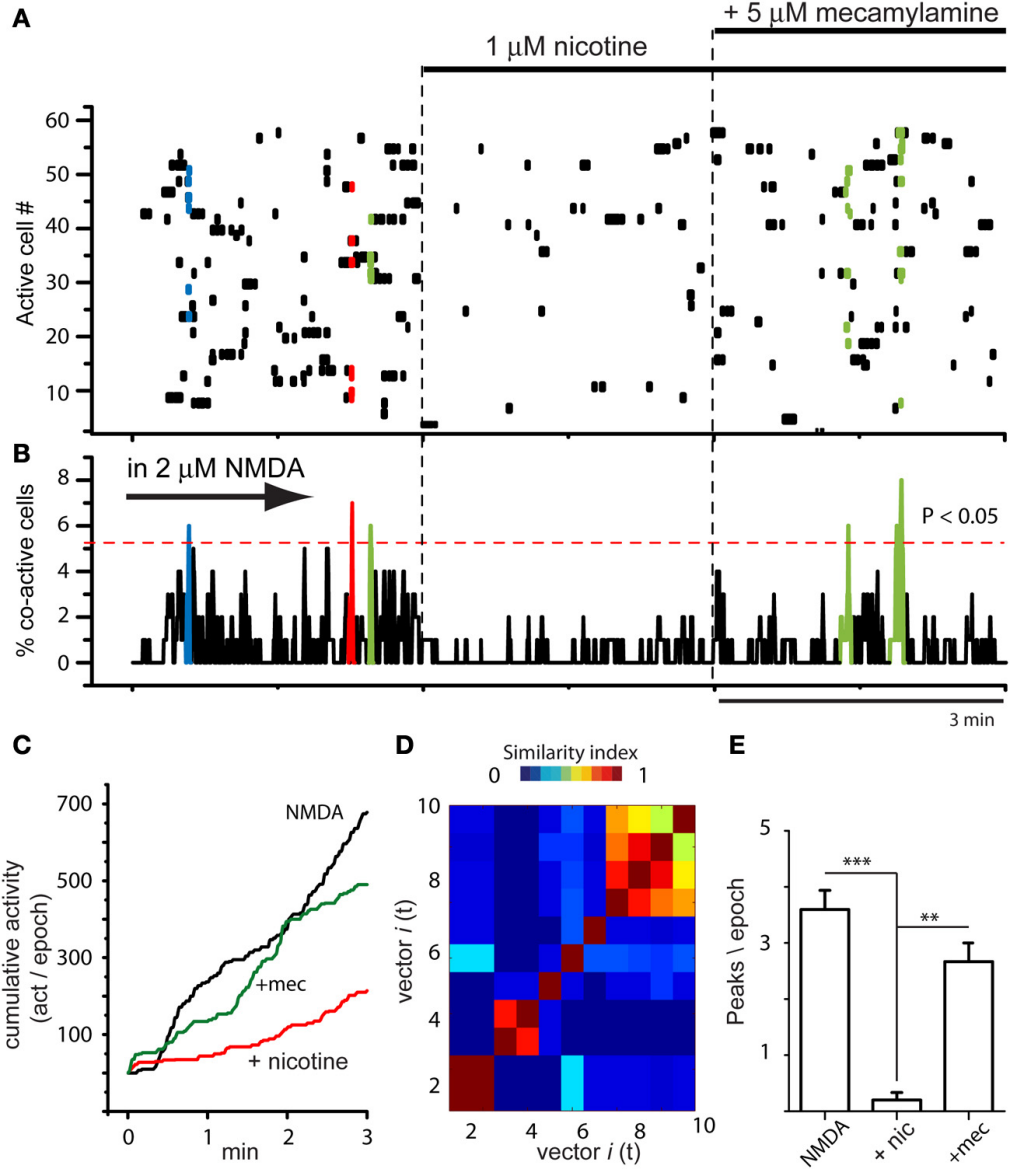
**Global actions of nicotine in the striatal circuit are blocked by mecamylamine. (A)** Raster plot showing circuit activity in the presence of NMDA. Similarly to Figure 2A, left epoch shows activity with spontaneous synchronization of different neuron pools (colored dots). Middle epoch: nicotine (1 μM) added to the bath saline in the presence of NMDA induced a decrease in activity and the absence of color indicates that significant events of spontaneous synchronization are now absent. Right epoch: addition of 10 μM mecamylamine allowed the return of some peaks of synchronization showing that the activity of nicotine was receptor dependent (*n* = 3 slices; 6 epochs). **(B)** Activity histogram displaying multicellular activity from the raster plot above. Significant peaks of synchronization (colored) appear during NMDA. They disappeared when nicotine was added and reappeared after addition of mecamylamine to the bath saline. **(C)** Cumulative activity taken from histogram in **(B)** shows that rate of accumulated activity was significantly lower in the presence of nicotine plus NMDA than with NMDA alone. It is also shown that mecamylamine virtually restored the level of accumulated activity over time in the continuous presence of both NMDA and nicotine. **(D)** Matrix of vectors similarity along time reassured that activity in NMDA is due to network activity. **(E)** Histogram showing that the number of peaks of synchronization per epoch decreased significantly when nicotine was added in the presence of NMDA-induced activity (^***^P < 0.001) and then increased again, significantly (^**^P < 0.01), when mecamylamine was added in the presence of nicotine.

Figure 3 shows that actions of nicotine were blocked by 10 μM mecamylamine, a non-selective and non-competitive antagonist of nAChrs (*n* = 4). NMDA produced the usual increase in circuit activity with spontaneous and statistically significant peaks of synchronization (Figures 3A,B left epoch; NMDA was present during the whole experiment). This activity was greatly decreased when nicotine was added to the bath (middle epoch in Figures 3A,B), when significant peaks of synchronization disappeared, denoting that many individual neurons stopped firing (dots in the matrix). However, they partially returned when mecamylamine was added in the presence of both NMDA and nicotine (Figures 3A,B right epoch), suggesting that the actions of nicotine were receptor specific. A more complete characterization of the nicotinic receptors involved is out of the scope of the present report. Cumulative plots (Figure 3C) show that circuit activity is significantly lowered only when nicotine was added: an average of the sample of experiments yields 163 ± 1.3 (act/min) in NMDA, which decays to 51 ± 0.7 (act/min) during nicotine (*n* = 3; average significance of *P* < 0.01). Note that subsequent addition of mecamylamine produced a return in the tendency of cumulative activity (green trace), even in the continuous presence of nicotine. The vectors similarity matrix reassured that NMDA-induced activity had assembly dynamics (Figure 3D). Histogram in Figure 3E shows average of synchronization peaks taken from several control slices in the presence of NMDA: 3.6 ± 0.34 peaks per epoch (*n* = 10 slices and *n* = 10 epochs). This average decreased significantly when nicotine was added to the superfusion: 0.2 ± 0.13 peaks per epoch (^***^P < 0.001; *n* = 10 slices and *n* = 10 epochs). Note that when the non-selective nAChr antagonist, mecamylamine, was added to the bath saline, the peaks of synchronization returned gradually and significantly: 2.7 ± 0.33 peaks per epoch (^**^P < 0.01; *n* = 4 slices; *n* = 6 epochs).

In summary, nicotine was capable to reduce circuit activity in the striatal network and its actions were both reversible (Figure 2) and blocked by mecamylamine (Figure 3). But nAChrs are ionotropic channels that carry inward currents that should excite, not inhibit circuit activity, just as NMDA-receptors. How it is possible that two ionotropic cationic receptors have opposite actions in the same circuit? According with some cell-focused studies, we hypothesized that the actions of nicotine were indirect via the activation of striatal GABAergic interneurons (e.g., English et al., 2011; Ibáñez-Sandoval et al., 2011; Luo et al., 2013). To see if this latter hypothesis was true we tried to block nicotine effects on the circuit with GABA_A_-receptor antagonists.

### Inhibitory action of nicotine in the striatal circuit is blocked by GABA_A_-receptor antagonists

**Figure 4:**
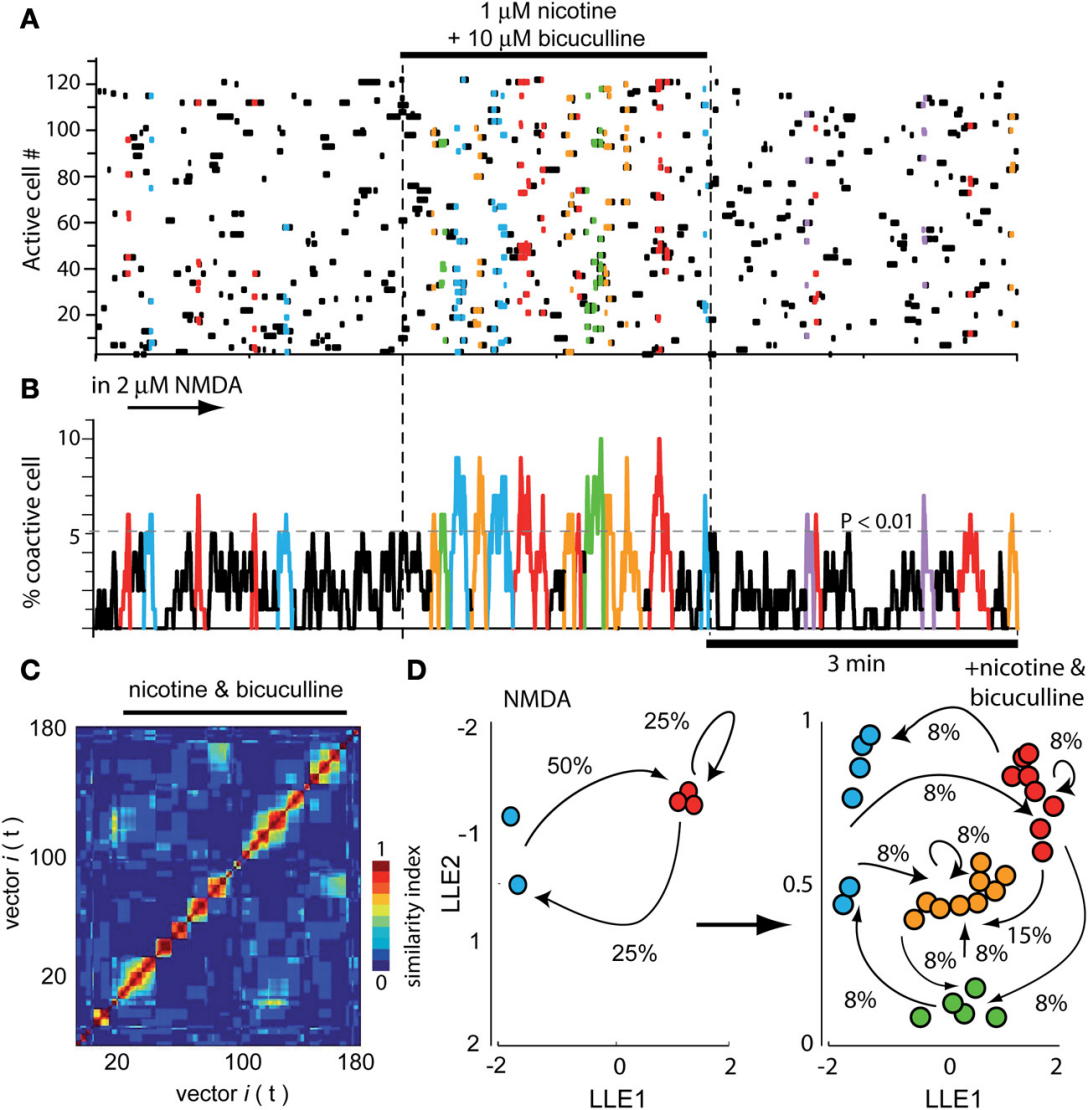
**Global actions of nicotine in the striatal circuit are blocked by bicuculline. (A)** Raster plot showing again three different epochs of activity. At the left epoch, the typical activity of the striatal circuit with 2 μM NMDA is seen displaying two neuron pools with synchronized activity (red and blue). Middle epoch: neuronal activity is dramatically increased due to the addition of 1 μM nicotine plus 10 μM bicuculline into the bath saline. In contrast with what happens when bicuculline is administered alone (Carrillo-Reid et al., 2008), nicotine plus bicuculline showed a quite diverse set of synchronization peaks. Right epoch: nicotine plus bicuculline actions were reversible. **(B)** Activity histogram of summed multicellular activity showing that in control (left) there are five peaks of spontaneous synchronization belonging to two different neuron pools (blue and red). Peaks of synchronization increase in number and classes when both nicotine and bicuculline are added to the bath (middle epoch). When bicuculline is given alone, circuit activity is manifested by a highly recurrent peak of synchronization (Carrillo-Reid et al., 2008). Right epoch: activity begins to return to normal after the drugs were washed off. **(C)** Similarity matrix in the presence of both drugs. **(D)** LLE before (NMDA left) and after addition of nicotine plus bicuculline (right): note more network states and more transitions in circuit activity when both drugs are present.

Figure 4A illustrates a raster plot with three epochs (separated by dashed vertical lines). At left, the usual neuronal activity found in striatal tissue after addition of 2 μM NMDA in the bath is shown (cf., Figure 1). Significant peaks of synchronization are present (Figure 4B left epoch; blue and red). At the middle epoch, 1 μM nicotine plus 10 μM bicuculline were added. In contrast with the action of bicuculline alone, where the activity of the circuit increases using the same pool of neurons over and over again (see Figure 8 in Carrillo-Reid et al., 2008 and Figure 5 in Jáidar et al., 2010), bicuculline together with nicotine, generated an activity of the circuit that displayed an increase of synchronization peaks (Figures 4A,B middle epoch). In these conditions, nicotine did not restrain circuit activity anymore and the induced activity consisted in a frenzy sequence of peaks coming from different neuron pools that alternate their activity without pace. 10 μM gabazine had the same results when administered with nicotine (*n* = 3; not shown but see: López-Huerta et al., 2012, 2013). Alternations among neuronal pools became frequent and rarely repeated.

In summary, bicuculline added alone increased activity based in a single or dominant peak of synchronization (see Carrillo-Reid et al., 2008; Jáidar et al., 2010), nicotine added alone, decreased the activity and peaks of synchronization disappeared (Figure 2), but nicotine added with bicuculline increased activity based on an increase in the peaks of synchronization (Figure 4; cf., middle epoch with left and right epochs). The wash off of both drugs returned the circuit to usual levels in terms of activity during NMDA (Figures 4A,B right epoch). Similarity matrix includes vectors activity with bicuculline and nicotine (Figure 4C). Dimensionality reduction LLE compares circuit dynamics before (Figure 4D left; only two states red and blue) and after nicotine plus bicuculline (Figure 4D right; four states). Note that transitions between network states drastically augmented. This action was surprising in the sense that it suggests that several different sets of neurons are being activated by nicotine in spite of blocking GABA_A_-receptor transmission, as in the case of DA D_2_-receptor agonist action in the striatal circuit (cf., Figure 5B in Carrillo-Reid et al., 2011).

### Parkinsonian activity in dopamine depleted striatum is reduced by nicotine

A mice sample was lesioned unilaterally with 6-OHDA. The degree of DA deprivation in the striatal tissue was tested provoking turning behavior 8 days after the surgery using automated rotometers and amphetamine (4 mg/kg, i.p.). Animals showing >500 turns ipsilateral to the injected side were considered for further experiments (López-Huerta et al., 2012).

**Figure 5:**
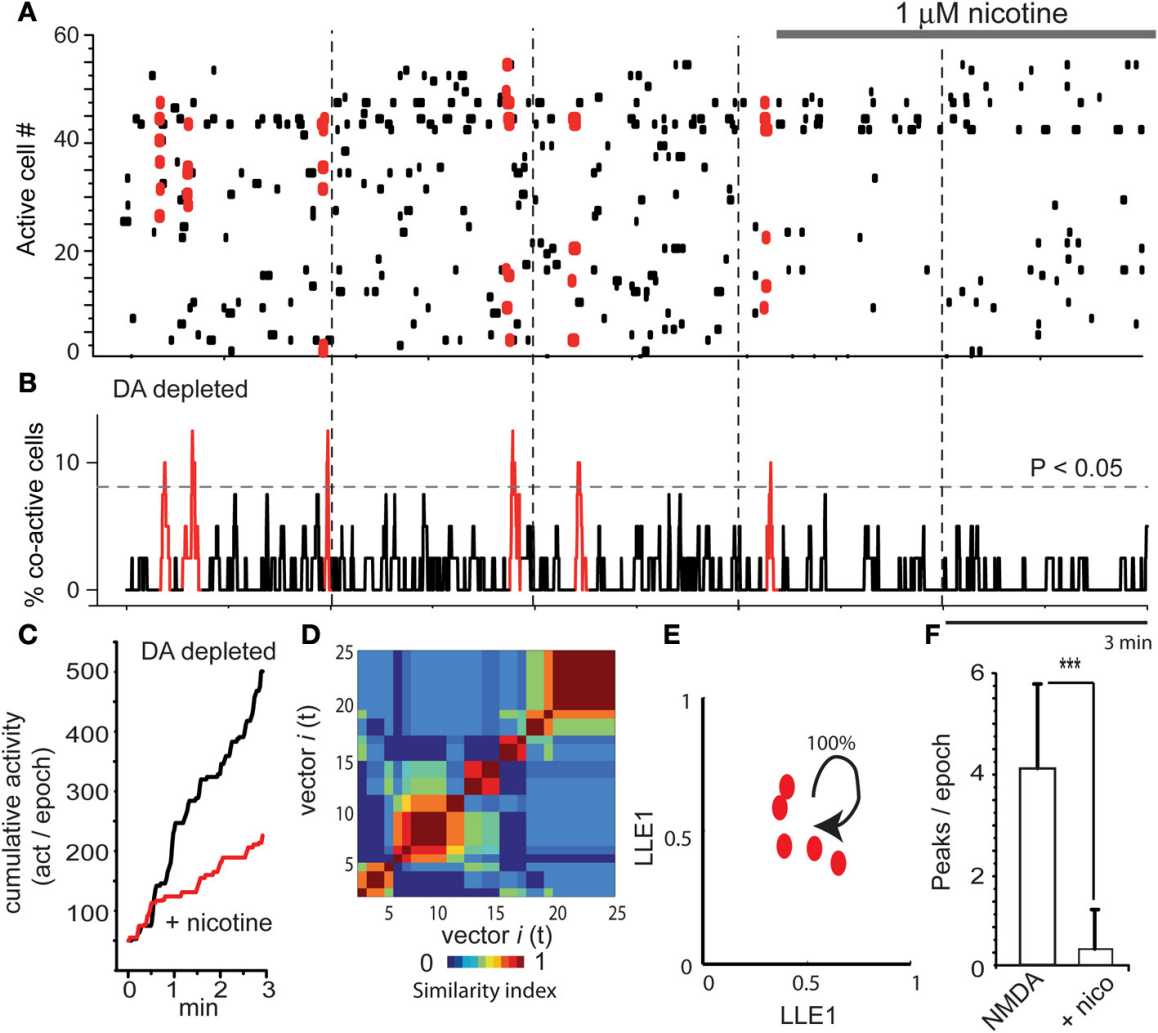
**Nicotine inhibits Parkinsonian activity in the dopamine depleted striatal microcircuit. (A)** Raster plots showing five epochs of activity in a DA-depleted striatal circuit. There is spontaneous neuronal activity in the absence of NMDA (first three epochs shows activity in a 6-OHDA rodent model of hemi-Parkinsonism, see Materials and Methods). However, significant synchronization is composed of the same neuron pool. Last two epochs show that nicotine reduces this activity. **(B)** Note that activity during DA deprivation exhibits significant peaks of synchronization. However, all are depicted with the same color since similarity indexes indicate that these vectors are built with a similar set of neurons presenting correlated activity in a recurrent mode. Note that these peaks disappear during nicotine. **(C)** Cumulative activity is significantly higher during DA-depletion than during DA-depletion with nicotine. **(D)** Similarity matrix shows similar neuronal vectors dominating the activity through the time in the DA depleted microcircuit. **(E)** LLE shows that the same network state keeps repeating its firing over time in the DA depleted tissue (all dots are of the same group-color), as though the circuit could not leave this dominant network state (Jáidar et al., 2010). Nicotine was capable to suppress this recurrent network state significantly. **(F)** The histogram shows that peaks of spontaneous synchronization are virtually abolished by nicotine. ^***^P < 0.005.

As already reported (Jáidar et al., 2010), the DA depleted striatum exhibited spontaneous activity and peaks of statistically significant synchronization that appear spontaneously in the absence of NMDA or any other excitatory drive (Figures 5A,B; first three epochs). This pathological activity is different to the one recorded in the control striatum without NMDA (leftmost epoch in Figure 1A). It is also different than NMDA-induced activity in control tissue (Carrillo-Reid et al., 2008; Jáidar et al., 2010). First three epochs in the raster plot of Figure 5A show Parkinsonian activity followed by addition of nicotine in the last two epochs. In fact, nicotine was added after the beginning of the fourth epoch to appreciate the quick action of nicotine. Activity histogram (Figure 5B) shows that the DA-depleted microcircuit presents significant peaks of synchronization. Note however, that in this case the peaks are composed by the same pool of neurons (same color red), having recurrent activity once and again (Jáidar et al., 2010) as it is the case with bicuculline actions when it is given alone (Carrillo-Reid et al., 2008). In other words, DA-depletion produces an increased activity with no alternation. Interestingly, addition of 1 μM nicotine to the bath saline (last two frames) abolished Parkinsonian activity and the peaks of synchronization (last two epochs in Figures 5A,B). Cumulative activity clearly shows more activity over time for the Parkinsonian microcircuit (Figure 5C): average rate of activity over time in the DA deprived circuit was: 170 ± 1 (act/min) while it was 54 ± 0.4 (act/min) after nicotine (*n* = 6; *P* < 0.006), showing that nicotine significantly reduced pathological activity.

As previously reported (Jáidar et al., 2010), peaks of synchronization were made by similar neuronal vectors or the same network state having recurrent activity, according to similarity index and LLE analysis (Figures 5D,E; Jáidar et al., 2010). Note LLE (Figure 5E) showing the same network state recurring on itself absorbing all synchronized neurons, suggesting that this is the microcircuit correlate of Parkinsonian slow oscillations. It was surprising that nicotine was capable to suppress the excess in activity and the dominant network state. Finally, we also quantified the number of synchronization peaks per epoch: it was an average of 4.2 ± 1.7 peaks/epoch (*n* = 6 slices; *n* = 18 epochs) in a sample of DA deprived slices and 0.3 ± 0.7 (peaks/epoch) in DA deprived slices during nicotine treatment (Figure 5F; *n* = 6 slices; *n* = 12 epochs; ^***^P < 0.005). In fact, the action of nicotine in this early Parkinsonian tissue is as potent as that of L-DOPA (cf., Plata et al., 2013).

## Discussion

Global action of nicotine in the striatal microcircuit reduced both the NMDA-induced neuronal activity in control tissue and the Parkinsonian pathological activity in the rodent 6-OHDA model of PD. However, nicotine, as NMDA, is an agonist of ligand-gated ionotropic channels whose role is to generate inward currents and depolarize target neurons. Nevertheless, each one of these agonists, NMDA and nicotine, has a completely different and even opposed action in the striatal circuit.

Cell-focused studies have disentangled an array of different actions of nicotine at the cellular and synaptic levels. First, nicotine activates nAChrs in incoming glutamate terminals inducing glutamate release (e.g., Marchi et al., 2002; Zhang and Warren, 2002; Campos et al., 2010). If this action were the predominant action at the microcircuit level, then it would be facilitatory of circuit activity and add to the activation produced by NMDA in the control circuit or to the pathological activity found in the DA-depleted circuit. This was not the case.

Second, nicotine also activates dopaminergic synapses inducing DA release (e.g., Wonnacott et al., 2000; Grady et al., 2007; Keath et al., 2007; Livingstone and Wonnacott, 2009; Xiao et al., 2009; Cachope et al., 2012; Threlfell et al., 2012). This action taken alone would lead to the induction of some kind of activity in the network (Carrillo-Reid et al., 2011) although this activity would be unpredictable solely based in cell-focused studies. On the one hand, DA induces firing in striatal interneurons. This action would favor inhibition of the circuit (Bracci et al., 2002). Nevertheless, besides postsynaptic activation of interneurons, DA also inhibits the release of GABA from the terminals of the same or different interneurons (Centonze et al., 2003). This, apparently, is a contradictory result. The end result of both actions taken together is hard to infer with cell-focused studies alone. This fact supports the need to observe actions of transmitters at the network level, and not only at the cellular level. Also, when seen at the circuit level, both classes of DA receptors, D_1_ and D_2_, induce an increase in spontaneous synchronized activity (Carrillo-Reid et al., 2011). Besides, individual SPNs may be inhibited or excited by DA (e.g., Kiyatkin and Rebec, 1996; Carrillo-Reid et al., 2011) depending on context. Taken one by one, it is difficult to make sense of all these actions and any inference about the end results is precluded. This fact supports the direct study of the global action in the microcircuit as a whole.

Furthermore, in case the activity of the circuit is increased due to DA-depletion (Jáidar et al., 2010), collateral inhibition among SPNs is decreased, and most actions of nicotine would be on GABAergic synapses made by interneurons. In such a case, DA analogs, such as L-DOPA, completely restore the control activity of the circuit, dramatically and reversible, being this action a neuronal correlate of behavioral and clinical trials (Plata et al., 2013). Accordingly, one question of the present work is how much nicotinic actions approximate that of L-DOPA.

Third, nAChrs actions have also been recorded in striatal GABAergic interneurons during cell-focused studies: nicotinic activation of several interneuron types such as fast-spiking (FS), low-threshold spiking (LTS), tonically firing neurogliaform cells, and others, release GABA upon nicotinic activation and inhibit projection neurons (Koós and Tepper, 2002; Wilson, 2004; Kreitzer and Malenka, 2008; Livingstone and Wonnacott, 2009; Xiao et al., 2009; English et al., 2011; Ibáñez-Sandoval et al., 2011; Luo et al., 2013). As the cell-focused studies described above, this appears to be another important action of nicotine when observed in isolation, cell by cell.

To conclude, cell-focused studies have revealed a host of mixed and parallel actions, some pointing to inhibitory and others to excitatory types of activity. Taken together, all these actions make hard to foretell what would be the global action of nicotinic receptors activation on the striatal microcircuit as a whole. The importance of answering this question is that clinical studies claim improvements of motor and cognitive symptoms in PD patients subjected to nicotine analogs (Fagerström et al., 1994; Villafane et al., 2007), that is, after systemic administration. Moreover, some epidemiological studies claim less incidence of PD in smokers (Gorell et al., 1999; Herman et al., 2001; Driver et al., 2009). Therefore, it is logical to question about what is the predominant action of nicotine in the striatal circuit when tonic concentrations are raised. To observe the answer to this matter directly at the microcircuit level, some type of multicell recording and therefore, a more sophisticated analysis, are required. However, the results surpassed any expectation about which of the mixed and parallel actions described in cell-focused studies predominates: nicotine readily and reversibly decreased control (with NMDA) and Parkinsonian activity. This observation gives raise to perhaps more important questions: what is the relation between nicotine actions and its supposed anti-Parkinsonian activity? Could it be used as an adjunct therapy in PD?

In summary, we demonstrate that the predominant action of a tonic elevation in nicotine concentration in the striatal microcircuit is the inhibition of network activity through the activation of GABAergic transmission, since inhibitory nicotinic action was blocked by GABA_A_-receptor antagonists. Secondly, the inhibitory activity could be either on the assembly dynamics induced by an uncorrelated excitatory drive such as NMDA (Carrillo-Reid et al., 2008), or else, on the pathological activity derived from DA depletion (Jáidar et al., 2010) in early Parkinsonian animals models.

In the second case, nicotinic action is a microcircuit correlate of the already described anti-Parkinsonian actions of nicotine (Fagerström et al., 1994; Gorell et al., 1999; Costa et al., 2001; Herman et al., 2001; Villafane et al., 2007; Driver et al., 2009; Quik et al., 2007; García-Montes et al., 2012; Kawamata et al., 2012). Indeed, in comparison to our early Parkinsonian animal models (Jáidar et al., 2010), we show that the action of nicotine is almost as strong as that of L-DOPA (Plata et al., 2013). Such a strong anti-Parkinsonian action at the circuit level was certainly unexpected.

Many classes of striatal interneurons are known to express nAChrs and are capable to be activated by nicotinic analogs (Koós and Tepper, 2002; Wilson, 2004; Quik et al., 2007; Kreitzer and Malenka, 2008; Livingstone and Wonnacott, 2009; Xiao et al., 2009; English et al., 2011; Ibáñez-Sandoval et al., 2011; Luo et al., 2013). As a result, they release GABA and inhibit SPNs. This inhibition is blocked by GABA_A_-receptor antagonists such as bicuculline (e.g., English et al., 2011; Ibáñez-Sandoval et al., 2011; Luo et al., 2013). Accordingly, here it is shown that global inhibitory nicotinic actions could be blocked with GABA_A_-receptor antagonists such as bicuculline suggesting that the strong decrease in circuit activity, normal or pathological, is due to massive interneurons activation. In fact, the action of bicuculline alone in the circuit has been reported both in control activity (with NMDA; Carrillo-Reid et al., 2008) and in Parkinsonian activity (with DA-depletion; Jáidar et al., 2010). When administered alone, bicuculline produces an increase in activity, but similarly to Parkinsonian activity it is characterized by a dominant pool of neurons having spontaneous synchronization in a recurrent way (Carrillo-Reid et al., 2008). In the DA-depleted tissue, activity entrenches the dominant state produced by DA absence destroying the alternating dynamics that may remain (Jáidar et al., 2010). In contrast, when bicuculline was given in the presence of nicotine, a plural set of peaks of synchronization appeared, manifesting a strong assembly dynamics with abundant trajectories in the LLE plot. This behavior suggests that several classes of interneurons are being activated.

Further research is needed to find out which of the interneuron classes predominate over the others, since each element of the circuit may express a different nAChr. The importance of this future dissection about the mechanism of how these powerful nicotinic action may happen is that it is known that PD course with hypercholinergia (rev in: Pisani et al., 2005; Goldberg and Reynolds, 2011) and that some types of interneurons become hyperexcitable during DA depletion (Dehorter et al., 2009), a result that appear as counter-intuitive given the present data. Therefore, this action may involve the activation of a specific interneuron network (with a specific nAChr-class). A strong candidate is the neurogliaform interneuron which releases abundant GABA setting the stage for volume transmission (Ibáñez-Sandoval et al., 2011). Alternatively, nicotine may favor the synchronized network activity of an interneuron network united by gap junctions (Tepper et al., 2010). In both cases, activation of these neurons may be capable to inhibit the interneurons that are overactive during PD (Dehorter et al., 2009), including perhaps, the cholinergic ones. This future research may find that a specific receptor is involved, a necessary step to find selective ligands with potential therapeutic use. Finally, *in vivo* experiments are needed to answer another question: how such a potent inhibition would allow common motor tasks.

In any case, the present results highlight the possibility of using nicotine analogs as an adjunct to L-DOPA in PD therapy.

The increase in DA release induced by nicotine may have its own beneficial actions as long as some DA-terminals still remain (Wonnacott et al., 2000; Grady et al., 2007; Keath et al., 2007; Livingstone and Wonnacott, 2009; Xiao et al., 2009; Cachope et al., 2012; Threlfell et al., 2012). A possible difficulty is that DA and glutamate release are tied together, and have a reciprocal interaction and regulation. In any case, here, we present a bio-assay that allows the evaluation of control and pathological activity of the striatal microcircuit in which the actions of drugs with suspected therapeutic actions may be tested and compared with usual behavioral assays.

## Conclusion

Calcium imaging techniques may serve to design bio-assays to test potential anti-Parkinsonian drugs in *in vitro* brain slices. Here, we tested nicotine, which has been suspected to possess anti-Parkinsonian activity for a long time. Indeed, it had an action similar to L-DOPA assayed in the same preparation (cf., Plata et al., 2013). However, both drugs were assayed in early Parkinsonian animals tested with turning behavior as a correlate of DA-deprivation. Further research is needed to test nicotine analogs in later stages of the disease, as for example, in dyskinesias.

In addition, we searched the mechanism of nicotinic actions which turned out to be indirect: the global effect of elevating the tonic concentration of nicotine in the striatal microcircuit was that of inhibiting the circuit in a way that was blocked by GABA_A_-receptor antagonists, that is, most probably by activating a set of inhibitory interneurons. Further research is needed to find out which neurons and nAChRs are involved in these actions. But of the many parallel and sometimes contradictory actions that have been described separately in cell-focused studies, the activation of interneurons appeared to be the predominant one.

## Author contributions

Víctor Plata, Mariana Duhne, Pavel Rueda-Orozco made most experiments, Ricardo Hernández-Martinez and Mariana Duhne lesioned animals with 6-OHDA and evaluated behavior, Víctor Plata and Jesús Pérez-Ortega made or modified acquisition and analysis software, Elvira Galarraga, René Drucker-Colín, and José Bargas had the original ideas, planned and reviewed the experiments, José Bargas and Víctor Plata wrote the article. Irakli Intskirveli reviewed the article.

### Conflict of interest statement

The authors declare that the research was conducted in the absence of any commercial or financial relationships that could be construed as a potential conflict of interest.
